# Identification of Novel Protein-Protein Interactions of *Yersinia pestis* Type III Secretion System by Yeast Two Hybrid System

**DOI:** 10.1371/journal.pone.0054121

**Published:** 2013-01-22

**Authors:** Huiying Yang, Yafang Tan, Tingting Zhang, Liujun Tang, Jian Wang, Yuehua Ke, Zhaobiao Guo, Xiaoming Yang, Ruifu Yang, Zongmin Du

**Affiliations:** 1 State Key Laboratory of Pathogen and Biosecurity, Beijing Institute of Microbiology and Epidemiology, Beijing, China; 2 Department of Clinical Medicine, College of Veterinary Medicine, Sichuan Agricultural University, Yaan, Sichuan, China; 3 State Key Laboratory of Proteomics, Beijing Proteome Research Center, Beijing Institute of Radiation Medicine, Beijing, China; George Mason University, United States of America

## Abstract

Type III secretion system (T3SS) of the plague bacterium *Y. pestis* encodes a syringe-like structure consisting of more than 20 proteins, which can inject virulence effectors into host cells to modulate the cellular functions. Here in this report, interactions among the possible components in T3SS of *Yersinia pestis* were identified using yeast mating technique. A total of 57 genes, including all the pCD1-encoded genes except those involved in plasmid replication and partition, pseudogenes, and the putative transposase genes, were subjected to yeast mating analysis. 21 pairs of interaction proteins were identified, among which 9 pairs had been previously reported and 12 novel pairs were identified in this study. Six of them were tested by GST pull down assay, and interaction pairs of YscG-SycD, YscG-TyeA, YscI-YscF, and YopN-YpCD1.09c were successfully validated, suggesting that these interactions might play potential roles in function of *Yersinia* T3SS. Several potential new interactions among T3SS components could help to understand the assembly and regulation of *Yersinia* T3SS.

## Introduction

Type III secretion system (T3SS) is one of the most important virulence mechanisms of the lethal plague pathogen, *Yersinia pestis*. Three human pathogenic bacteria in the genus of *Yersinia*, *Y. pestis, Y. enterocoiltica* and *Y. pseudotuberculosis* share a common T3SS encoded by a 70 kb virulence plasmid [1]. *Y. pestis* has caused three pandemics and often leads the patients to death if they are not treated in time, while the other two species merely cause limited gastrointestinal manifestations [2]. Despite of the great diversity in their pathogenesis and clinical outcomes, T3SS virulence mechanism is indispensable for all the three pathogens. T3SS is a virulence strategy that is widely distributed in Gram-negative bacterium. Upon the bacterial contact with the host cell, it can efficiently deliver virulent proteins called effectors into the host cell cytosol, where they hijack the host cells by modulating the singling pathway of host immune response [3], [4].

T3SS is hitherto one of the most complicated virulence mechanisms utilized by Gram-negative bacteria pathogens. In *Yersinia* T3SS, more than 20 proteins have been shown to participate in structure, function or regulation of this tightly regulated macromolecular machine [5]. Electromicroscopic observation of the core complex of T3SS, termed injectisome, isolated from *Shigella*, enteropathogenic *Escherichia coli*, *Samonellona* and *Yersinia* spp. have revealed a syringe-like structure consisting of a base body and a protruding needle [5], [6]. The base body composed of two pairs of rings, which embedded in the bacterial outer and inner membrane, respectively. The ring spanning the outer membrane (called OM ring) consists of ring-like oligomers of 12–14 YscC secretin monomers [7], [8]. Pilotin lipoprotein YscW is required for proper insertion of YscC into the bacterial outer membrane [9]. The ring in inner membrane (called MS ring) is made of YscJ lipoproteins, which are anchored to the inner membrane and form a specialized patch in membrane for recruiting putative inner membrane-embedded components such as YscR, YscS, YscT, YscU and YscV [10]. YscD is proposed to participate in MS ring formation and possibly connect the rings in the two membranes [11]. YscR, S, T, U and V built the export apparatus which supposed to be found within the inner ring [12]. At the cytosolic side of the injectisome, YscN ATPases provides the energy supply to facilitate the translocation of effectors by the T3SS machine [13]. YscN could interact with YscK and YscL, which play roles in regulation of YscN function [14], [15]. Previous studies suggested that the cytoplasmic ring (C ring) that is composed of YscQ [16] is beneath the MS ring in *Yersinia* injectsome. YscQ forms a platform at the cytoplasm-inner membrane interface for the recruitment of other proteins, including the ATPase [5]. The needle of the injectsome is made up of the polymerized YscF [17]. YscP acts as a molecule ruler to control the needle length of the injectisome. It was hypothesized that one end of YscP molecule is tethered at the end of base body and the other end to the tip of the growing needle, and the needle keeps growing until YscP is fully stretched [18], [19]. The tip structure of the needle is composed of YopB and YopD, which serves as a scaffold for the formation of a pore in the host cell membrane [20]. LcrV forms tip structure of injectisome together with YopB and YopD [21].

At least six effectors called Yops (*Yersinia* outer memberane proteins), YopE, YopH, YopM, YopT, YpkA/YopO, and YopJ/YopP, are ultimately translocated into eukaryotic cell through T3SS [22]. Chaperon proteins, including SycE, SycH, SycT, SycO, SycD and SycB, are small, acidic proteins that are highly conserved in structure, which can bind with their cognate proteins and keep them stable and competent for secretion [23], [24]. YopN, LcrG, TyeA, SycN, and YscB are demonstrated to play roles in Yops targeting translocation and control of the on-off switch of the secretion channel 25,26.

Identification of protein interactions among various components of T3SS helps to predict and elucidate the structures and functions of the secretion machine. Many protein interactions in T3SS have been identified and well characterized so far, resulting in the significant advance in the understanding of its function and structure. High throughput methods for investigation of protein-protein interactions, including yeast two hybrid (Y2H) techniques [14], surface plasmon resonance (SPR) and matrix-assisted laser desorption time-of-flight mass spectrometry (MALDI-TOF MS) [27] have been utilized to reveal potential new protein interactions within *Yersinia* type III secretion apparatuses. Jackson *et al.* had explored interactions among the Ysc proteins including YscEFGHIKLN and YscQ of *Y. pestis* using the Y2H system [14]. They revealed that YscL interacts with YscN and YscQ, and YscQ interacts with YscL and YscK, respectively, which were later validated both *in vivo* and *in vitro* by GST pull down and biochemical analysis [14]. Swietnicki *et al.* investigated the interactions between 15 proteins including Yops, regulators (YmoA), and chaperones using SPR and MALDI-TOF MS, and more than 20 novel protein interactions were identified [27]. However, only partial components of T3SS were included in these works and interactions beyond those proteins thus have not yet been explored. In this study, we cloned 57 genes encoded in pCD1 plasmid and analyze the potential protein interactions within *Yersinia* T3SS using yeast mating techniques. A total of 21 interactions were identified, among which 12 have not been previously reported. Some of the novel interactions were confirmed by GST pull down assay, and whether they play roles in the regulation, structure or function of T3SS need to be determined in future studies.

## Results

### Constructions of Baits and Preys of *Yersinia* T3SS Genes

According to the genome annotation of *Y. pestis* CO92, there are 96 ORFs in plasmid pCD1 and 45 of them encode proteins that are identified or predicted to be involved in T3SS [28]. In the rest of 51 ORFs, exclude the putative pseudogenes, transposase, plasmid replication and partitioning genes, there are 12 genes that are predicted to encode hypothetical proteins with unknown functions. These hypothetical proteins have the potential to participate in the structure or function of T3SS. Therefore, a total of 57 genes in plasmid pCD1, including 45 identified or predicted T3SS genes and the 12 unknown function genes, were selected in this study (Table S1). Primers incorporating *attB* recombination region were designed to amplify the target genes. All the amplified genes were cloned into pDONER 201 by Gateway recombination cloning method, then, transferred into pDEST32 and pDEST22 destination vectors to generate DB-ORF and AD-ORF fusions, respectively. The lengths of *lcrD* and *ypkA* gene are 2115 and 2196 bp, respectively, which are beyond the range of optimal length for Y2H mating screening. Therefore, *lcrD* was cloned as fragments pending 1–1500 bp and 618–2115 bp with an overlap region pending 618–1500 bp. *ypkA* was cloned as fragments pending 1–1500 bp and 385–2199 bp with an overlap region pending 385–1500 bp.

### Yeast Two-hybrid Mating Analysis of Protein-protein Interactions among the *Yersinia* T3SS Components

Competent yeast strain MaV203 (MATα) was transformed with BD-gene plasmids as baits and Mav103 (MATa) with AD-gene as prays. Each of the 59 prays was one-by-one mated with 59 baits in 96-well plate format for screening potential protein-protein interactions, which is designated as forward screening. In reverse direction, each of the 59 baits was one-by-one mated with 59 preys, which is designated as reverse screening. The forward screening and reverse screening constitute one round of Y2H mating assay. We conducted three rounds of screening in this study, and the results were shown in Table 1. The positive and negative controls were included on each selective plate, including SC-Leu-Trp plates for the selection of yeast cells transformed with both the bait and prey plasmids and other selective plates for the assessment of the reporter genes expression, to ensure that the obtained results are valid. Results will be discarded if the positive and the negative controls on a selective plate do not perform normally. Some of the interactions were screened out repeatedly in different rounds of experiment and totally 21 pairs of interactions were identified after the redundant interactions were removed. In all those interactions, 9/21 (42.8%) have been reported previously (Table S2), indicating that our Y2H screening has relative high reliability and can detect a portion of the well characterized interactions among *Yersinia* T3SS components. However, we also noted that even though we performed multiple rounds of Y2H mating assay, only about 1/3 of the known interactions among T3SS components could be detected. This indicates that the false negative rate is relative high too and some potential interactions could be missed in our Y2H screen. The identified interactions can be divided into three categories according to the functions of the involved proteins (Table 1). 1) Interactions among secretion regulatory components. 2) Interactions among the chaperones and secretion substrates. 3) Other types of interactions. Almost all the interactions belong to the first two categories have been reported previously.

**Table 1:** Protein-protein interactions of the *Y. pestis* TTSS measured byY2H.

Interaction type	Interactor A		Interactor B		Known	Round			
	Gene ID	Protein name	Gene ID	Protein name		1st	2nd	3rd	Times
Secretion regulatory	1.39c	YopN	1.38c	TyeA	Y	+	+	+	3
components	1.37c	SycN	1.51	YscB	Y	+	+	+	3
	1.32c	LcrG	1.31c	LcrV	Y	+	−	+	2
Chaperon and secretion	1.05c	SycE	1.05c	SycE	Y	+	−	−	1
substrates	1.95	SycH	1.62	YscM	Y	−	+	+	2
	1.95c	SycH	1.67c	YopH	Y	+	+	+	3
	1.95c	SycH	1.95c	SycH	Y	+	−	−	1
	1.54	YscE	1.56	YscG	Y	+	−	−	1
	1.57	YopR	1.95c	SycH		+	−	−	1
Other interactions	1.4	YscN	1.61	YscL	Y	+	+	−	2
	1.06	YopE	1.34c-2	LcrD		+	−	−	1
	1.56	YscG	1.3	SycD		−	+	−	1
	1.5	YscA	1.33c	LcrR		+	−	−	1
	1.56	YscG	1.38c	TyeA		+	−	−	1
	1.58	YscI	1.55	YscF		+	−	−	1
	1.33c	LcrR	1.73c	SycO		+	−	−	1
	1.46	YscT	1.08c	Hypothetical		+	−	−	1
	1.09c	Hypothetical	1.39c	YopN		+	−	−	1
	1.09c	Hypothetical	1.08c	Hypothetical		+	+	+	3
	1.16c	Hypothetical	1.38c	TyeA		+	+	+	3
	1.16c	Hypothetical	1.15c	Hypothetical		+	−	−	1
Total number	21 pairwise interactions Identified in different rounds					19	8	7	

It has long been known that all the Y2H system have the problem of false negatives and false positives. False negatives are the major source leading to the reproducible problem of Y2H [29]. Given these technique limitations and in order to gain interaction pairs as many as possible, we performed 3 rounds of screens in this study. We noted that results from different rounds of screens showed obvious inconstancy, and the first round of screen seems to give the best result since the identified interaction pairs in this round were the most, which contains lots of known interactions (Table 1). The low reproducibility of our Y2H results might be caused by the fluctuations of the experiment conditions or the transient/weak interactions involved. For instance, any variation in yeast cells transformation efficiency, the quality of the plasmids, the incubation time for yeast cells, or other factors on each stage of screen could influence the overall performance of the Y2H screen. Weak interactions and the transient interactions might be difficult to be detected with high reproducibility, which will contribute greatly to the inconstancy of the results. To ensure the best performance of Y2H screen, adequate measures and various controls should be adopted to guarantee the quality of each step of Y2H screen. Considering the false negative of Y2H is very high, and only a portion of known interaction have been detected, we deem that multiple rounds of screens followed by cautious validation of the interactions will be help for getting more information on the interactions among the interested proteins.

### Confirmation of Protein-protein Interactions by GST Pull Down Assay

**Figure 1:**
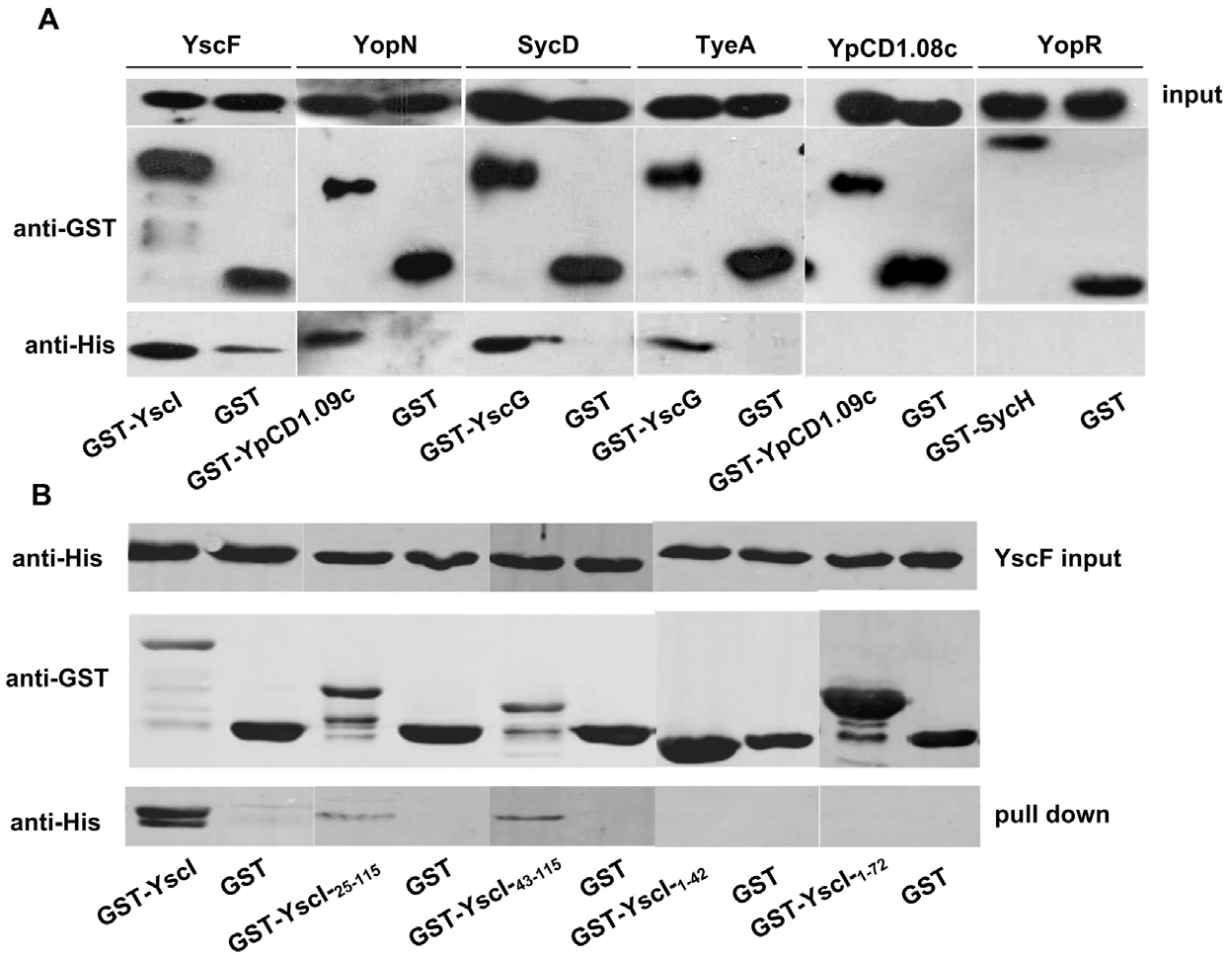
GST pull-down validation of novel interactions identified by yeast mating assay. GST pull-down results for six pairs of interaction are shown as labeled. Purified GST-tagged *Y. pestis* T3SS proteins were immobilized onto the glutathione sepharose beads 4B and incubated overnight with the putative interacting proteins that were identified by yeast mating assay. After extensive washing, the beads were added with 2×SDS buffer and boiled, and the proteins were separated by SDS-PAGE electrophoresis. The separated proteins were transferred onto the PVDF membrane followed by immunoblotting using anti-GST or anti-His antibodies. The top panel shows the His-tagged proteins that were loaded in each pull-down assay for GST or GST-tagged *Y. pestis* protein. The middle and the lower panels show the pull-down proteins.

To validate the interactions that were newly identified in this study, GST pull down approach were used. All the 18 *Yersinia* proteins that are involved in 12 pairs of novel interactions have been tried to be cloned and expressed in *E. coil* BL21. However, 5 proteins could not be expressed (LcrD, YscT and YpCD1.15c) or expressed in form of inclusion body (LcrR and YpCD1.16c) under all the experimental conditions we have tried (Table S3). Thus, a total of 14 proteins of *Y. pestis* were successfully expressed and purified, and available for the validation of 6 pairs of interactions by GST pull down assay (Table S4). Four of the six interactions were successfully validated, which include interactions of YscF-YscI, YopN-YpCD1.09c, SycD-YscG, and TyeA-YscG (Figure 1A). To further identify the regions in YscI that is important for the YscF binding ability, GST-tagged truncates of YscI were expressed in *E. coli* and determined for their abilities to bind with YscF. Results showed that the C-terminal domain of YscI that is highly conversed in the inner rod proteins of many other T3SS is important for the YscI-YscF interactions (Figure 1B), which provides valuable hints for further studies. For YpCD1.09c-YpCD1.08c and SycH-YopR interactions, we have conducted pull down assays repeatedly for several times but no positive result has been obtained. This suggested that interactions between these proteins were very weak or transiently occurred if they were not false positive results.

### Yops Secretion Analysis in *YpCD1.09* Mutant

**Figure 2:**
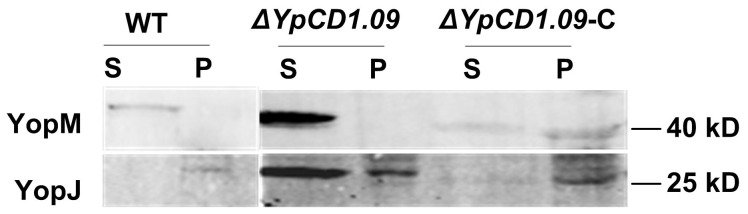
Analysis of YopJ and YopM secretions in the *ΔYpCD1.09* mutant. Bacterial strains were inoculated into TMH without calcium and incubated at 26°C to an OD600nm of 1.0. The bacterial cultures were then transferred to 37°C for 3 hours incubation to induce the Yops expression and secretion. Proteins from the culture supernatant (S) and the cell pellets (P) were separated by SDS-PAGE electrophoresis and detected using rabbit polyclonal antibodies against YopJ and YopM.

One of the characters of *Yersinia* T3SS is that the bacteria growth will be greatly restricted under the culture conditions that fully induce T3SS expression and Yops secretion, a phenomenon called low calcium response (LCR) [1]. Previous reports showed that *Yersinia* bacteria defective in some members of T3SS proteins showed abnormal LCR. To reveal the potential functional significance of YpCD1.09-YopN interaction, we constructed an YpCD1.09 deletion mutant, Δ*YpCD1.09*, using λ-Red based mutagenesis method [30]. Growth curve of the wild type strain and Δ*YpCD1.09* in TMH medium with and without calcium at 26°C or 37°C were determined, and the results showed that the low calcium response in Δ*YpCD1.09* was almost unaffected although the growth rate was a little slower for Δ*YpCD1.09* than that of the wild type strain (Figure S1, A). It has been shown that YopN can form YopN/SycN/YscB/TyeA complex and blocks Yops secretion under the Yops non-secretion condition [31]. We want to know whether YpCD1.09-YopN interaction has any impact on this function of YopN, an important regulator controlling when the Yops secretions are initiated. YopJ and YopM in the culture supernatants and the bacteria pellets from cultures of *ΔYpCD1.09* and the wild type strain were analyzed, and it was found that *ΔYpCD1.09* steadily secreted more YopJ and YopM than the wild type strain did when grown at 37°C in the absent of calcium (Figure S1, B). However, no YopJ and YopM secretion could be detected when grown in the culture medium supplemented with calcium, consisting with the growth curve of *ΔYpCD1.09*, which indicated a normal LCR phenotype (Figure S1, B). Furthermore, we confirmed that YopJ and YopM secretions in the *ΔYpCD1.09* complementary strain expressing YpCD1.09 protein were comparable with those secreted by the wild type strain (Figure 2). In summary, we assume that YpCD1.09 might inhibit the formation of the YopN/SycN/YscB/TyeA plunger through interacting with YopN, therefore YopJ and YopM secretion in *ΔYpCD1.09* were increased. However, YpCD1.09-YopN interaction seems to have no effect on the blocking of Yops secretion of bacteria under the non-secretion condition.

## Discussion

### Interactions between Chaperons and Secretion Substrates

Most of the identified interactions (5/6) in this category have been previously reported (Table 1). The only novel interaction is between SycH and YopR (YscH). However, we could not confirm this interaction by pull down assay (Figure 1). SycH belongs to a chaperon category whose members interact with multiple secreted substrates. It can assist the transport of YopH and regulatory components of the T3SS, YscM1 and YscM2 in enteropathogenic *Yersinia spp*, or LcrQ in *Y. pestis* [32], [33], [34]. The SycH binding sites in different cognates have conserved N-terminal residues. Deletion analyses localized the binding site for SycH in YopH to residues 17∼70 [33], which corresponds to the translocation signal. YopR is located in the operon of *yscABCDEFGHIJKLM.* Little is known about the function of YopR other than it is secreted into the extracellular milieu during the early stages of infection and it contributes to virulence [35]. Our result suggested that YopR might be a new member that can be specifically recognized by SycH, and binding to SycH might facilitate the secretion of YopR into the extracellular environment. However, further investigation needs to be done to determine the significance of our findings, since we cannot validate this interaction by GST pull down assay.

### Interactions between Secretion Regulatory Components

All the identified interactions belong to this category have been previously reported (Table S2), which comprise SycN-YscB, YopN-TyeA and LcrG-LcrV interactions [31], [36]. Secretion of effectors by T3SS is inhibited before the close contact with the host cells *in vivo*, or when cultured under secretion non-permission condition *in vitro*. Interestingly, all these 3 interactions play critical roles in the negative regulation on the secretion of effectors. SycN and YscB interact with one another and form a SycN/YscB complex that can bind to an N-terminal region of YopN, which is necessary for the stable expression and for efficient YopN secretion [36]. However, SycN or YscB alone cannot bind to YopN [36]. TyeA binds to a C-terminal domain of YopN and has an inhibitory effect on YopN secretion [25], [26]. Thus, YopN protein, the SycN/YscB chaperon and TyeA could form a YopN/SycN/YscB/TyeA complex that is required to block the Yops secretions under the non-secretion permission conditions. Crystal structures of YopN in complex with its heterodimeric chaperone SycN/YscB and the co-regulatory protein TyeA had been reported [31]. Consisting with the previous reports, we detected the SycN-YscB and YopN-TyeA interactions in yeast mating assay, while interactions of YscB-YopN or SycN-YopN were not. LcrG blocks the Yop secretion apparatus from the cytoplasmic side under the non-secretion condition [37]. When the secretion inhibitory signals are removed, the increasing amount of LcrV form 1∶1 LcrG-LcrV complex, leading to the remove of the secretion block to fully induce Yop and LcrV secretion and expression [38].

### Others Interactions

#### LcrD-YopE interaction

LcrD belongs to a protein family that participates in the surface presentation or secretion of the virulence determinants [39]. The secretion and transcriptional regulation of a number of Yops and LcrV depends on the functional LcrD protein [40]. The secondary structures of LcrD protein family members show significant similarities and contain a hydrophobic amino-terminal half that consist of 6–8 potential transmembrane domains and a hydrophilic carboxy terminus which is predicted to reside in the cytoplasm [39]. LcrD membrane topology analysis showed that 18–347 amino acids residues construct 8 amino-terminal transmembrane domains that anchor a large cytoplasmic carboxyl-terminal domain to the inner membrane. Two LcrD baits expressing 1–500 and 207–705 amino acids residues, respectively, were constructed and used to perform the yeast mating analysis in this study. LcrD_(207–705)_ bait that expresses the complete cytoplasmic domain was found to interact with YopE, whereas the LcrD_(1–500)_ could not, suggesting that the interaction between YopE and LcrD might be mediated by the cytoplasmic domain. We found it was difficult to purify soluble recombinant LcrD_(207–705)_ protein to perform further GST pull down assays to validate the interaction between LcrD and YopE. This result hints that YopE might interact with LcrD at the inner membrane of bacteria, and we suspect that this interaction might have roles in the sequential secretion of Yop effectors.

#### YscF-YscI interaction

Polymers of YscF protein construct the needle of *Yersinia* injectisome [41]. The inner rod that is composed of YscI is a cylindrical structure that traverses the base of injectisome and connects the needle to the basal side of the base body [42], [43]. Marlovits et al. proposed a mechanism on how substrate specificity of *S. typhimurium* SPI-1 injectisome switches from the subunits of inner rod and needle proteins to the secretion substrates [6]. They showed that the needle composed of PrgI (YscF homolog) and the inner rod composed of PrgJ (YscI homolog) assemble simultaneously, and the termination of the inner rod leads to conformational changes on the cytoplasmic side of the base body, resulting in the initiation of secretion of effectors. Similarly, it has been showed that the formation of inner rod is critical for substrate specificity switching in *Yersinia* T3SS, and YscP and YscU play central roles in this process through controlling the secretion of YscI inner rod protein [43]. YscI mutants containing point mutations of Q84A, L87A, and L96A secreted substantial amount of Yops yet exhibit severe defects in YscF needle formation. [43]. Interestingly, interaction between YscF and YscI was identified in our yeast mating analysis and the results of GST pull down assays with the GST-tagged YscI truncates indicated that the C-terminal of YscI is critical for YscI-YscF interaction. We hypothesize that YscI might play its role on secretion of YscF protein through direct interaction with it, which could provide a potential explanation that YscI mutants displays defect in the substrate specificity switching from YscF to Yop effectors [43].

#### Hypothetical proteins

We obtained some interactions between the hypothetical proteins and the known T3SS components. For instance, we found that YpCD1.16c interacted with TyeA, a component of negative regulator complex of *Yersinia* T3SS, and YpCD1.09c interacted with YopN. We also detected some interactions between the hypothetical proteins, such as interactions pairs of YpCD1.09c-YpCD1.08c and YpCD1.16c -YpCD1.15c. The locations of the encoding genes of these interacting proteins are neighbors to each other, implying that they might have functional relevant. Furthermore, GST pull down assay results validated that YopN does interact with YpCD1.09c *in vitro*; Yops secretion analysis showed that more YopJ and YopM were secreted in the *ΔYpCD1.09* under the Yops secretion inducing condition, suggesting a regulatory role of YpCD1.09 in *Yersinia* T3SS. These results hint that these hypothetical proteins might be new members of T3SS.

### Conclusions

**Figure 3:**
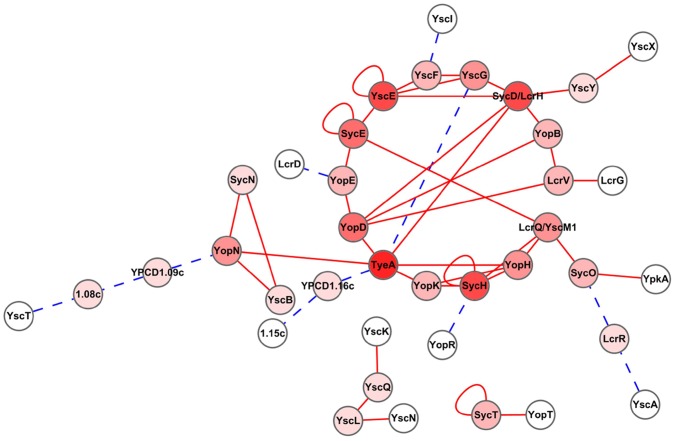
Graphic view of the protein-protein interaction network of *Yersinia* T3SS. The graphic view of the network was drawn using Cytoscape 2.0 software [47]. All the functionally well characterized interactions among *Yersinia* type III secretion apparatus components and the potential novel interactions identified in this study were included. The darker colors indicate the higher centrality of a protein in this network. Red lines indicate known interactions that had been reported and functionally validated by biochemical methods. Blue and dotted lines indicate novel interactions identified by our yeast mating assays.

**Figure 4:**
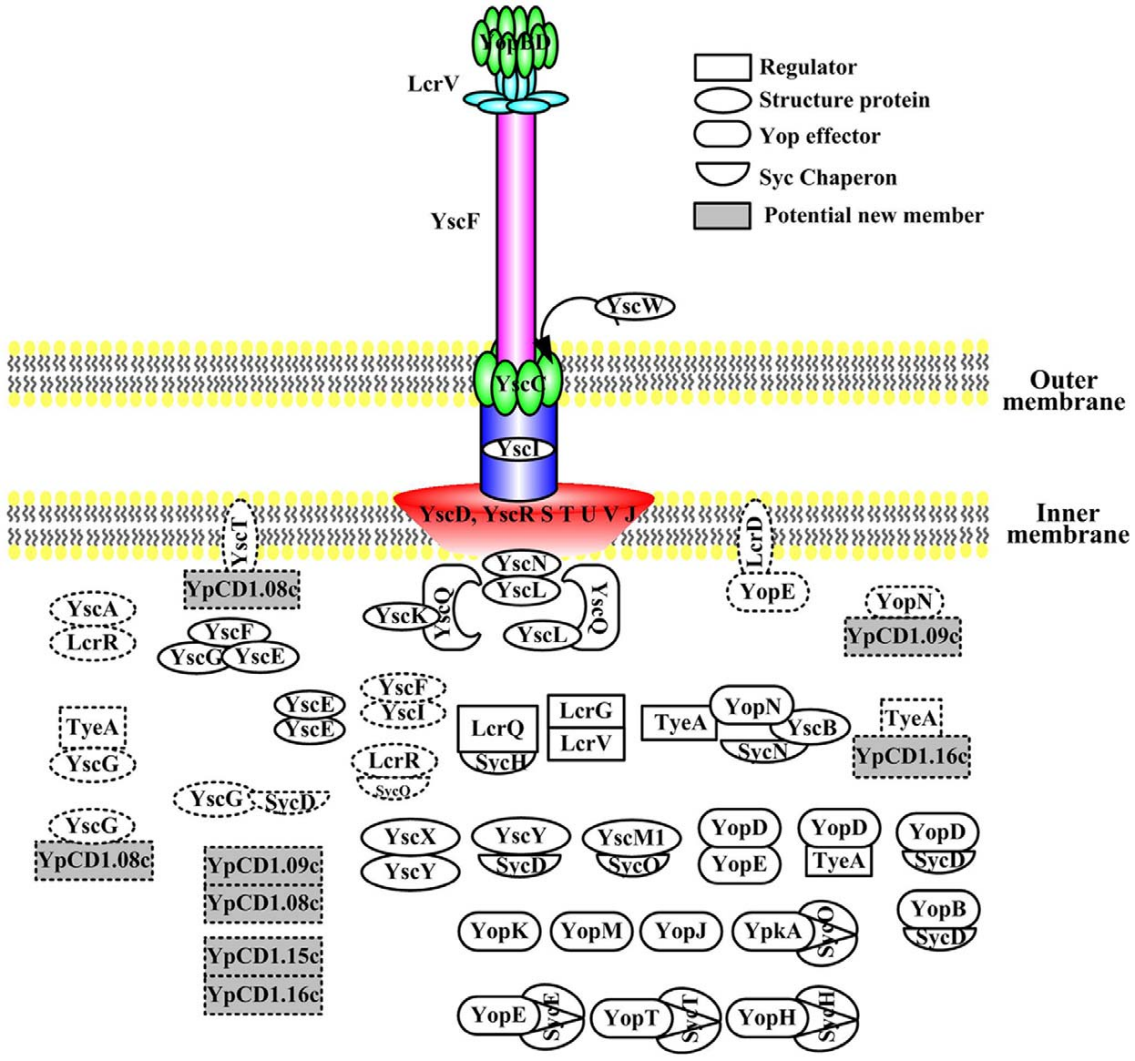
Schematic representation of *Yersinia* type III secretion apparatus and the protein-protein interactions among the different components. Symbols in dotted line indicate that interactions between these proteins have not been previously reported and were newly identified in this study.

Based on the previously published protein-protein interactions between T3SS components (Table S2) and the interactions identified in this study (Table 1), we construct an interaction network of *Yersinia* T3SS. This network comprise of 51 interactions among 38 T3SS proteins (Figure 3). Each node represents a protein and the lines between the nodes indicate that the two connected proteins interact with each other. The darker colors of a node indicate the higher centralities of a protein in this network, which means there are more proteins interacting with this node. We found that chaperons tend to be highly connected. For instance, SycD and SycH interact with as many as 6 proteins in this network, implying the central position of these proteins in the network. This suggests that chaperons are not only small proteins to facilitate the translocation of the cognate effectors (SycH is chaperon for YopH) or translocators (SycD is chaperon for YopB and YopD) but also major nodes in the T3SS regulatory network, which might regulate the T3SS from the injectisome assembly to Yops secretion process. Schematic diagram structure of *Yersinia* T3SS is showed in Figure 4, and all the protein-protein interactions that have been well characterized are labeled according to the locations of corresponding proteins in the type III secretion apparatus. The novel interactions identified in this study are showed by symbols in dotted line. Only interactions that have been validated by biochemical or functional analysis are displayed in this schematic diagram, and interactions that were identified by Y2H, MS or SPR methods yet not validated by an independent method or functionally proved are not included. This regulatory network will become more comprehensive and complete along with the identification of new interactions between T3SS protein members.

## Materials and Methods

### Bacterial and Yeast Strains, Plasmids, and Culture Conditions

*Y. pestis* strain 201 was grown at 28°C in heart infusion broth (BHI) or on Luria–Bertani (LB) agar plates. *Escherichia coli* DH5α and *E. coli* BL21 were routinely grown at 37°C in LB medium or on LB agar plates. In growth curve experiments, bacterial strains were cultivated in TMH medium supplemented or not supplemented with calcium. Growth curve experiments were performed as previously described [44]. Appropriate antibiotics were added if the cultivated strains harbor the plasmids with antibiotics marker. *Saccharomyces cerevisiae* strains MaV203 and MaV103 were grown at 30°C in YPD medium or on yeast minimal synthetic dropout (SD) agar plates. Gateway vectors pDONOR201, pDEST22, pDEST32 (Invitrogen) were used to construct yeast expressing plasmids, and pDEST17, pHGGWA (gift form Prof. Moras D.) [45] and pTH27 (Invitrogen) were used to construct expressing plasmids of the *Y. pestis* proteins in *E. coli*.

### DNA Manipulations

Gateway recombination cloning technology was used to construct the recombinant plasmids. PCR primers incorporating *attB* recombination sites followed by ORF-specific sequences were designed to amplify each target gene using genomic DNA of *Y. pestis* strain 91001 (biovar microtus) as template. The amplified products were cloned into pDONER 201 using BP Clonase enzyme mix (Invitrogen), and the presence of correct inserts was confirmed by DNA sequencing. Target genes in entry clones were subsequently inserted into pDEST32 and pDEST22 destination vectors to generate pEXP32 recombinants expressing DB (Gal4 DNA-binding domain)-ORFs and pEXP22 recombinants expressing AD (Gal4 activation domain)-ORFs, respectively. pEXP32 and pEXP22 constructs were individually transformed into haploid MaV203 *MAT*α and MaV103 *MATa* yeast strains, generating DB-ORF library and AD-ORF library, respectively. The target genes of *Y. pestis* (Table S1) were cloned into pDEST17 or pHGGWA destination vectors by LR to construct the plasmids expressing His- or GST-tagged proteins. After the sequences of the inserts were verified by DNA sequencing, the constructs were transformed into *E. coli* BL21 (DE3) for protein expression. *YpCD1.09* mutant strain was constructed using λ-red based mutagenesis strategy as previously described [44].

### Yeast Mating Experiment

MaV203 *MAT*α clones of DB-ORF library were arrayed in 96-well microtiter plates on solid YPDA medium. Baits in AD-ORF library which active *HIS3*, *URA3* and *lacZ* reporter genes were eliminate form Y2H mating by self-activation experiments. The positive control was MAV203 containing p C97-*Fos* and pC86-*Jun* plasmids (Invitrogen), which will show the strong interaction and the negative control was MAV203 containing pC97 and pC86 empty vectors (Invitrogen). For interaction mating, each AD-ORF was individually mated to all the BD-ORFs arrayed in 96-well microtiter plates. The yeast mixtures were grown in YPDA plate and allowed to be incubated at 30°C for 36 h. After mating, the clones were plated on SC-Leu-Trp plates by replica-plating for the selection of diploids. After additional incubation for 48 h, diploids cells were then examined for β-galactosidase activity phenotypes by replica-plating onto YPDA medium plate with a filter paper to perform the assay, as well as on SC-Leu-Trp-His+25 mM 3-AT plates and SC-Leu-Trp-Ura to assess elevated expression levels of the three two-hybrid phenotypes (β-galactosidase, His and Ura) [46]. Yeast mating procedure was carried out for the second time and all the procedures were the same with the first run except that AD-ORF transformed MaV103 cells were arrayed onto 96-well microtiter plates on solid YPDA, and BD-ORFs were mated to the AD-ORFs on the 96-well microtiter plates. The former screening called forward screening, and the latter screening called reverse screening. We performed multiple screenings, each including forward and reverse screening each time, to identify interactions within *Yersinia* T3SS as far as possible.

### Recombinant Proteins Expression and Purification

BL21λDE3-harboring pDEST17 or pHGGWA constructs expressing *Yersinia* proteins were grown in LB broth at 37°C and induced with 1 mM IPTG. Bacterial cells expressing His-tagged proteins were resuspended in lysis buffer (50 mM NaH_2_PO_4_, pH 8.0, 300 mM NaCl, 10 mM imidazole) and lysed by sonication. The soluble 6× His-tagged proteins were separated from insoluble cell debris by centrifugation at 14 000× g and purified by affinity chromatography on Ni-NTA agarose (Qiagen). For purification of GST-tagged proteins, bacterial cells were resuspended in 1×PBS and lysed as above, and the proteins were purified with glutathione sepharose beads 4B (GE Healthcare) according to the standard protocol.

### GST Pull Down Assay

Pull-down experiments were performed with the purified recombinant proteins. Briefly, about 0.1 mg of GST-tagged recombinant proteins were incubated with glutathione sepharose 4B beads (50 µl slurry) for 4 h at 4°C. Beads were then washed thoroughly with PBS for 3 times and added with the His-tagged proteins (0.1 mg in PBS), followed by incubation at 4°C overnight. After washing, bound proteins were analyzed by SDS-PAGE and immunoblotting using anti-GST (TIANGEN) and anti-His (Sigma) monoclonal antibodies. Purified GST protein was incubated with each of the His-tagged proteins and subjected to the same procedures to serve as a negative control.

### Detection of Yops Secretions

Secretions of YopJ and YopM were detected as previously described [44]. Bacterial strains were grown in TMH medium with or without calcium at 26°C to an OD600 of about 1.0, and then transferred to 37°C for 3 h to induce the expression and secretion of Yops. The culture medium and the bacterial cells were separated by centrifugation and the cell pellets were weighted. To ensure the equal loading of each samples, a volume of 2×SDS-PAGE loading buffer was added into the collected bacterial cell pellets according to the proportion of 50 µl loading buffer per 10 mg bacterial cells. Proteins in culture supernatants were precipitated with ice-cold 10% trichloroacetic acid (TCA) on ice at 4°C overnight. After the pH was adjusted to 7.0 by adding 5 µl of Tris-HCl buffer (pH 8.9), the TCA-precipitated proteins were dissolved in half volume of 2×SDS-PAGE loading buffer that was added into the corresponding cell pellets. Proteins were separated by SDS-PAGE electrophoresis and detected using rabbit polyclonal antibodies against YopJ and YopM.

## Supporting Information

Figure S1 **Mutation of** ***YpCD1.09*** **gene does not influence the LCR.**
(TIF)

Table S1 **57 target genes encoded in pCD1 plasmid according to the genome annotation of** ***Y. pestis*** **CO92.**
(PDF)

Table S2 **Known interactions of** ***Y. pestis*** **T3SS components reported in literatures.**
(PDF)

Table S3 **List of 19** ***Y. pestis*** **T3SS proteins that were successfully cloned or expressed in this study.**
(PDF)

Table S4 **Twelve novel interactions identified in this study and the validation results by GST pull down assay were shown.**
(PDF)
