# Supplementary figures and images for: Identification of Novel Protein-Protein Interactions of Yersinia pestis Type III Secretion System by Yeast Two Hybrid System
Source: PLoS One. 2013 Jan 22;8(1):e54121. doi: 10.1371/journal.pone.0054121 (PMC3551969; doi:10.1371/journal.pone.0054121)

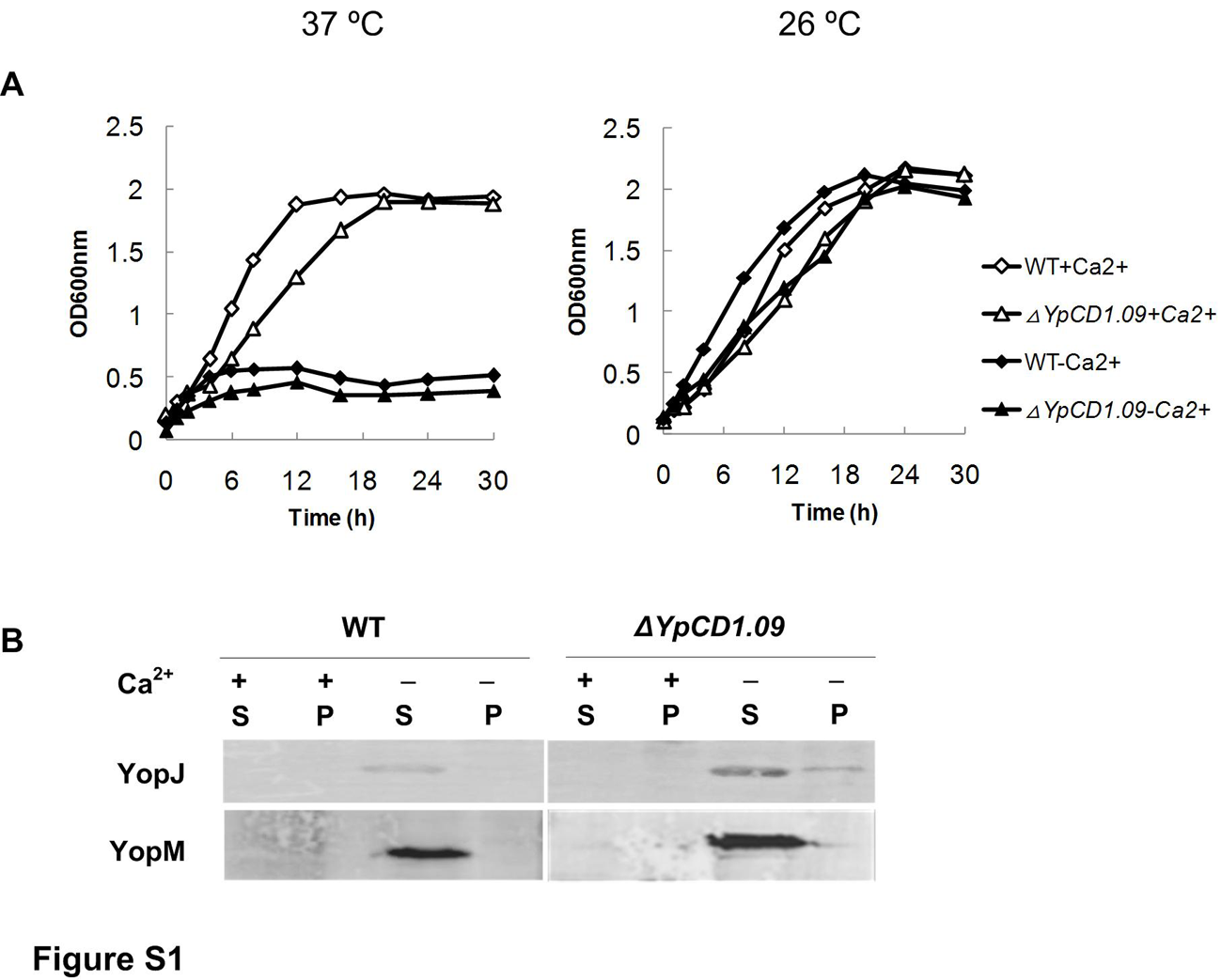

Supplement: Figure S1 — Mutation of YpCD1.09 gene does not influence the LCR. (TIF) [file pone.0054121.s001.tif]
