# Supplementary material for: Identification of Novel Protein-Protein Interactions of Yersinia pestis Type III Secretion System by Yeast Two Hybrid System
Source: PLoS One. 2013 Jan 22;8(1):e54121. doi: 10.1371/journal.pone.0054121 (PMC3551969; doi:10.1371/journal.pone.0054121)
Supplement: Table S1 — 57 target genes encoded in pCD1 plasmid according to the genome annotation of Y. pestis CO92. (PDF) [file pone.0054121.s002.pdf]

Table S1. 57 target genes encoded in pCD1 plasmid according to the genome annotation of *Y. pestis* CO92

Gene ID	Length aa	Gene name	product
YPCD1.05c	130	*yer A,sycE*	putative yopE chaperone sycE, yerA, yopE targeting protein
YPCD1.06	219	*yopE*	putative outer membrane virulence protein yopE
YPCD1.07	98		hypothetical protein
YPCD1.08c	99		hypothetical protein
YPCD1.09c	91		hypothetical protein
YPCD1.15c	152		hypothetical protein
YPCD1.16c	107		hypothetical protein
YPCD1.19c	182	*yopQ,yopK*	putative virulence determinant protein, yopK, yopQ
YPCD1.20	322	*yopT*	putative cytotoxic effector protein, yopT
YPCD1.21	132	*sycT*	putative yopT chaperone, sycT
YPCD1.22c	129		hypothetical protein , Y0063
YPCD1.23	139		hypothetical protein, Y0062
YPCD1.26c	409	*yopM*	probable targeted effector protein , yopM
YPCD1.28c	306	*yopD*	putative Yop negative regulation/targeting component, yopD
YPCD1.29c	401	*yopB*	putative Yop targeting protein, yopB
YPCD1.30c	168	*sycD,lcrH*	putative yopB/yopD chaperone, lcrH, sycD
YPCD1.31c	326	*lcrV*	putative V antigen, antihost protein/regulator, lcrV
YPCD1.32c	95	*lcrG*	putative Yop regulator, lcrG
YPCD1.33c	146	*lcrR*	hypothetical protein lcrR, lcrR
YPCD1.34c	704	*lcrD*	putative membrane-bound Yop protein, lcrD, yscV
YPCD1.35c	114	*yscY*	putative type III secretion protein, yscY
YPCD1.36c	122	*yscX*	putative type III secretion protein, yscX
YPCD1.37c	123	*sycN*	putative type III secretion protein, sycN
YPCD1.38c	92	*tyeA*	putative Yop secretion and targeting protein, tyeA
YPCD1.39c	293	*lcrE*	putative membrane-bound Yop targeting protein, yopN, lcrE
YPCD1.40	439	*yscN*	putative Yops secretion ATP synthase, yscN
YPCD1.41	154	*yscO*	putative type III secretion protein, yscO
YPCD1.42	455	*yscP*	putative type III secretion protein, yscP
YPCD1.43	307	*yscQ*	putative type III secretion protein, yscQ
YPCD1.44	217	*yscR*	putative Yop secretion membrane protein, yscR
YPCD1.45	88	*yscS*	putative type III secretion protein, yscS
YPCD1.46	261	*yscT*	putative type III secretion protein, yscT
YPCD1.47	354	*yscU*	putative type III secretion protein, yscU
YPCD1.48	131	*yscW*	putative Yop targeting lipoprotein, virG
YPCD1.49	271	*lcrF,virF*	putative thermoregulatory protein, lcrF, virF
YPCD1.50	32	*yscA*	hypothetical protein, yscA
YPCD1.51	137	*yscB*	hypothetical protein, yscB
YPCD1.52	607	*yscC*	putative type III secretion protein, yscC
YPCD1.53	419	*yscD*	putative type III secretion protein, yscD
YPCD1.54	66	*yscE*	putative type III secretion protein, yscE
YPCD1.55	87	*yscF*	putative type III secretion protein, yscF
YPCD1.56	115	*yscG*	putative type III secretion protein, yscG
YPCD1.57	165	*yscH, yopR, lc*	putative type III secretion protein, yscH, yopR, lcrP
YPCD1.58	115	*yscI, lcrO*	putative type III secretion protein, yscI, lcrO
YPCD1.59	244	*yscJ, ylpB*	putative type III secretion lipoprotein, yscJ, ylpB
YPCD1.60	209	*yscK*	putative type III secretion protein, yscK
YPCD1.61	221	*yscL*	putative type III secretion protein, yscL
YPCD1.62	115	*yscM, lcrQ*	putative type III secretion regulatory protein, yscM, lcrQ

YPCD1.67c	468	*yopH*	putative protein-tyrosine phosphatase Yop effector, yopH
YPCD1.68c	68		conserved hypothetical protein
YPCD1.71c	288	*yopP, yopJ*	putative targeted effector protein, yopP, yopJ
YPCD1.72c	732	*ypkA*	putative targeted effector protein kinase, ypkA
YPCD1.73c	149		hypothetical protein
YPCD1.82c	135		hypothetical protein
YPCD1.83c	107		hypothetical protein
YPCD1.91	109		hypothetical protein
YPCD1.95c	141	*sycH*	putative yopH targeting protein, sycH
