# Supplementary material for: Identification of Novel Protein-Protein Interactions of Yersinia pestis Type III Secretion System by Yeast Two Hybrid System
Source: PLoS One. 2013 Jan 22;8(1):e54121. doi: 10.1371/journal.pone.0054121 (PMC3551969; doi:10.1371/journal.pone.0054121)
Supplement: Table S2 — Known interactions of Y. pestis T3SS components reported in literatures. (PDF) [file pone.0054121.s003.pdf]

Table S2. Known interactions of *Y. pestis* T3SS components reported in literatures.

Gene ID	Protein nam	Gene ID	Protein name	References	Method
YPCD1.05c	SycE	YPCD1.06	YopE	Wattiau and Cornelis 1993	Affinity blot
YPCD1.95c	SycH	YPCD1.67c	YopH	Wattiau, Bernier et al. 1994	Affinity blot
YPCD1.30c	SycD/LcrH	YPCD1.28c	YopD	Wattiau, Bernier et al. 1994	Affinity blot
YPCD1.32c	LcrG	YPCD1.31c	LcrV	Nilles, Williams et al. 1997	cross-linking and copurification
YPCD1.37c	SycN	YPCD1.51	YscB	Day and Plano 1998	Protein cross-linking, immunoprecipitation, and yeast two-hybrid analyses
YPCD1.37c/1.51	SycN/YscB complex	YPCD1.39c	YopN	Day and Plano 1998	Y3H
YPCD1.38c	TyeA	YPCD1.39c	YopN	Iriarte, Sory et al. 1998	Copurification
YPCD1.38c	TyeA	YPCD1.28c	YopD	Iriarte, Sory et al. 1998	Copurification
YPCD1.31c	LcrV	YPCD1.29c	YopB	Sarker, Neyt et al. 1998	Copurification
YPCD1.31c	LcrV	YPCD1.28c	YopD	Sarker, Neyt et al. 1998	Copurification
YPCD1.28c	YopD	YPCD1.06	YopE	Hartland 1998	Affinity blot
YPCD1.21	SycT	YPCD1.20	YopT	Iriarte and Cornelis 1998	Biochemical method
YPCD1.30c	SycD/LcrH	YPCD1.29c	YopB	Neyt and Cornelis 1999	GST pull down
YPCD1.29c	YopB	YPCD1.28c	YopD	Neyt and Cornelis 1999	GST pull down
YPCD1.56	YscG	YPCD1.54	YscE	Day, Guller et al. 2000	Y2H,biochemical method
YPCD1.36c	YscX	YPCD1.35c	YscY	Day and Plano 2000	Y2H,affinity blot
YPCD1.40	YscN	YPCD1.61	YscL	Jackson 2000	Y2H
YPCD1.61	YscL	YPCD1.43	YscQ	Jackson 2000	Y2H
YPCD1.60	YscK	YPCD1.43	YscQ	Jackson 2000	Y2H
YPCD1.05c	SycE	YPCD1.05c	SycE	Wattiau and Cornelis 1993	Crystal structure
YPCD1.35c	YscY	YPCD1.30c	SycD/LcrH	Francis, Lloyd et al. 2001	Y2H
YPCD1.95c	SycH	YPCD1.95c	SycH	Neumayer, Groll et al. 2004	Crystal structure and biochemical method
YPCD1.62	LcrQ/YscM	YPCD1.95c	SycH	Cambronne and Sorg 2004	Gel filtration chromatography
YPCD1.62	LcrQ/YscM	YPCD1.05c	SycE	Swietnicki, O'Brien et al. 200	SPR
YPCD1.05c	SycE	YPCD1.54	YscE	Swietnicki, O'Brien et al. 200	SPR/MS
YPCD1.95c	SycH	YPCD1.19c	YopK	Swietnicki, O'Brien et al. 200	SPR/MS
YPCD1.30c	SycD/LcrH	YPCD1.54	YscE	Swietnicki, O'Brien et al. 200	SPR/MS
YPCD1.30c	SycD/LcrH	YPCD1.38c	TyeA	Swietnicki, O'Brien et al. 200	SPR/MS
YPCD1.38c	TyeA	YPCD1.19c	YopK	Swietnicki, O'Brien et al. 200	SPR/MS
YPCD1.38c	TyeA	YPCD1.67c	YopH/N	Swietnicki, O'Brien et al. 200	SPR/MS
YPCD1.67c	yopH/N	YPCD1.19c	YopK	Swietnicki, O'Brien et al. 200	SPR/MS
YPCD1.62	YscM1/N-te	YPCD1.67c	YopH/N	Swietnicki, O'Brien et al. 200	MS
YPCD1.54	YscE	YPCD1.54	YscE	Phan, Austin et al. 2005	Crystal structure
YPCD1.21	SycT	YPCD1.21	SycT	Buttner, Cornelis et al. 2005	Crystal structure
YPCD1.73c	SycO	YPCD1.72c	YpkA	Letzelter, Sorg et al. 2006	Size-exclusion chromatography
YPCD1.73c	SycO	YPCD1.62	YscM1	Dittmann, Schmid et al. 2007	Native gel electrophoresis

YPCD1.73c	SycO	YPCD1.73c	SycO	Dittmann, Schmid et al. 2007	Analytical gel filtration and chemical cross-linking
YPCD1.55	YscF	YPCD1.56	YscG	Ping Sun, Joseph E. Tropea et	Crystal structure
YPCD1.55	YscF	YPCD1.54	YscE	Ping Sun, Joseph E. Tropea et	Crystal structure

Birtalan, S. and P. Ghosh (2001). "Structure of the Yersinia type III secretory system chaperone SycE." Nat Struct Biol **8**(11): 974-978.

Buttner, C. R., G. R. Cornelis, et al. (2005). "Crystal structure of Yersinia enterocolitica type III secretion chaperone SycT." Protein Sci **14**(8): 1993-2002.

Cambronne, E. D. and J. A. Sorg (2004). "Binding of SycH chaperone to YscM1 and YscM2 activates effector yop expression in Yersinia enterocolitica." Journal of Bacteriology **186**(3): 829-841.

Day, J. B., I. Guller, et al. (2000). "Yersinia pestis YscG protein is a Syc-like chaperone that directly binds yscE." Infection and Immunity **68**(11): 6466-6471.

Day, J. B. and G. V. Plano (1998). "A complex composed of SycN and YscB functions as a specific chaperone for YopN in Yersinia pestis." Mol Microbiol **30**(4): 777-788.

Day, J. B. and G. V. Plano (2000). "The *Yersinia pestis* YscY protein directly binds YscX, a secreted component of the type III secretion machinery." J Bacteriol **182**(7): 1834-1843.

Dittmann, S., A. Schmid, et al. (2007). "The Yersinia enterocolitica type three secretion chaperone SycO is integrated into the Yop regulatory network and binds to the Yop secretion protein YscM 1." BMC Microbiology

Hartland, E. L. (1998). "In vitro association between the virulence proteins, YopD and YopE, of Yersinia enterocolitica." FEMS Microbiology Letters **162**: 207-213.

Iriarte, M. and G. R. Cornelis (1998). "YopT, a new Yersinia Yop effector protein, affects the cytoskeleton of host cells." Mol Microbiol **29**(3): 915-929.

Iriarte, M., M. P. Sory, et al. (1998). "TyeA, a protein involved in control of Yop release and in translocation of Yersinia Yop effectors." The EMBO Journal **17**(7): 1907-1918.

Jackson, M. W. (2000). "Interactions between type III secretion apparatus components from Yersinia pestis detected using the yeast two - hybrid system." FEMS Microbiology Letters **186**: 85-90.

Letzelter, M., I. Sorg, et al. (2006). "The discovery of SycO highlights a new function for type III secretion effector chaperones." The EMBO Journal **25**(13): 3223-3233.

Neumayer, W., M. Groll, et al. (2004). "Yersinia enterocolitica type III secretion chaperone SycH. Recombinant expression, purification, characterisation, and crystallisation." Protein Expr Purif **35**(2): 237-247.

Neyt, C. and G. R. Cornelis (1999). "Role of SycD, the chaperone of the Yersinia Yop translocators YopB and YopD." Mol Microbiol **31**(1): 143-156.

Nilles, M. L., A. W. Williams, et al. (1997). " *Yersinia pestis* LcrV forms a stable complex with LcrG and may have a secretion-related regulatory role in the low-Ca2+ response." J Bacteriol **179**(4): 1307-1316.

Phan, J., B. P. Austin, et al. (2005). "Crystal structure of the Yersinia type III secretion protein YscE." Protein Sci **14**(10): 2759-2763.

Ping Sun, Joseph E. Tropea, et al. (2008). "Structural characterization of the Yersinia pestis type III secretion system needle protein YscF in complex with its heterodimeric chaperone YscE/YscG." Journal of Molecular

Sarker, M. R., C. Neyt, et al. (1998). "The Yersinia Yop virulon: LcrV is required for extrusion of the translocators YopB and YopD." Journal of Bacteriology **180**(5): 1207-1214.

Swietnicki, W., S. O'Brien, et al. (2004). "Novel protein-protein interactions of the *Yersinia pestis* type III secretion system elucidated with a matrix analysis by surface plasmon resonance and mass spectrometry." J Biol

Wattiau, P., B. Bernier, et al. (1994). "Individual chaperones required for Yop secretion by Yersinia." Microbiology **91**: 10493-10497.

Wattiau, P. and G. R. Cornelis (1993). "SycE, a chaperone-like protein of Yersinia enterocolitica involved in the secretion of YopE." Mol Microbiol **8**(1): 123-131.

Francis, M. S., S. A. Lloyd, et al. (2001). "The type III secretion chaperone LcrH co-operates with YopD to establish a negative , regulatory loop for control of Yop synthesis in Yersinia pseudotuberculosis." Mol

Edqvist, P. J., J. Olsson, et al. (2003). "YscP and YscU regulate substrate specificity of the Yersinia type III secretion system." J Bacteriol **185**(7): 2259-2266.
