# Supplementary material for: Identification of Novel Protein-Protein Interactions of Yersinia pestis Type III Secretion System by Yeast Two Hybrid System
Source: PLoS One. 2013 Jan 22;8(1):e54121. doi: 10.1371/journal.pone.0054121 (PMC3551969; doi:10.1371/journal.pone.0054121)
Supplement: Table S3 — List of 19 Y. pestis T3SS proteins that were successfully cloned or expressed in this study. (PDF) [file pone.0054121.s004.pdf]

Table S3. List of 19 *Y. pestis* T3SS proteins that were successfully cloned or expressed in this study.

Gene ID	Protein name	Cloned	Soluble expressed	Tag Types
YpCD1.46	YscT	Y	-	
YpCD1.15c	Hypothetical	Y	-	
YpCD1.34c-2	LcrD	Y	-	
YpCD1.16c	Hypothetical	Y	N	
YpCD1.33c	LcrR	Y	N	
YpCD1.38c	TyeA	Y	Y	His
YpCD1.30	SycD/LcrH	Y	Y	His
YpCD1.55	YscF	Y	Y	His
YpCD1.56	YscG	Y	Y	GST
YpCD1.57	YopR	Y	Y	His
YpCD1.58	YscI	Y	Y	GST
YpCD1.38c	TyeA	Y	Y	His
YpCD1.39c	YopN	Y	Y	His
YpCD1.08c	Hypothetical	Y	Y	His
YpCD1.09c	Hypothetical	Y	Y	GST
YpCD1.73c	SycO	Y	Y	His
YpCD1.95c	SycH	Y	Y	GST
YpCD1.06	YopE	Y	Y	His
YpCD1.50	YscA	Y	Y	His

Note:

"-" indicates protein that could not be expressed in this study.
