# Supplementary material for: Identification of Novel Protein-Protein Interactions of Yersinia pestis Type III Secretion System by Yeast Two Hybrid System
Source: PLoS One. 2013 Jan 22;8(1):e54121. doi: 10.1371/journal.pone.0054121 (PMC3551969; doi:10.1371/journal.pone.0054121)
Supplement: Table S4 — Twelve novel interactions identified in this study and the validation results by GST pull down assay were shown. (PDF) [file pone.0054121.s005.pdf]

Table S4. Twelve novel interactions identified in this study and the validation results by GST pull down assay were shown.

Gene ID	Protein name	Soluble expressed	Gene ID	Protein name	Soluble expressed	GST-pull down
YpCD1.34c-2	LcrD	-	YpCD1.06	YopE	Y	NA
YpCD1.15c	Hypothetical	-	YpCD1.16c	Hypothetical	N	NA
YpCD1.46	YscT	-	YpCD1.08c	Hypothetical	Y	NA
YpCD1.5	YscA	Y	YpCD1.33c	LcrR	N	NA
YpCD1.33c	LcrR	N	YpCD1.73c	SycO	Y	NA
YpCD1.16c	Hypothetical	N	YpCD1.38c	TyeA	Y	NA
YpCD1.56	YscG	Y	YpCD1.30	SycD/LcrH	Y	Y
YpCD1.56	YscG	Y	YpCD1.38c	TyeA	Y	Y
YpCD1.58	YscI	Y	YpCD1.55	YscF	Y	Y
YpCD1.09c	Hypothetical	Y	YpCD1.39c	YopN	Y	Y
YpCD1.09c	Hypothetical	Y	YpCD1.08c	Hypothetical	Y	N
YpCD1.95c	SycH	Y	YpCD1.57	YopR	Y	N

Notes:

"-" indicates protein that could not be expressed in this study.

"NA" indicates interaction that could not be analyzed by GST-pull down assay.
